# Medium Chain Triglycerides Modulate the Ketogenic Effect of a Metabolic Switch

**DOI:** 10.3389/fnut.2020.00003

**Published:** 2020-01-31

**Authors:** Camille Vandenberghe, Valérie St-Pierre, Mélanie Fortier, Christian-Alexandre Castellano, Bernard Cuenoud, Stephen C. Cunnane

**Affiliations:** ^1^Research Center on Aging, Sherbrooke, QC, Canada; ^2^Nestlé Health Science, Lausanne, Switzerland; ^3^Department of Medicine, Université de Sherbrooke, Sherbrooke, QC, Canada

**Keywords:** medium chain triglycerides, ketone, glucose, insulin, free fatty acids

## Abstract

Ketones provide an alternative brain fuel and may be neuroprotective in older people. Little is known of how to optimize the ketogenic effect of C8:0–C10:0 medium chain triglyceride supplement (kMCT). Metabolic switching (MS) from glucose to ketones as a fuel may have metabolic benefits but has not been extensively studied in humans. The objective of the present study was to use an 8 h metabolic study day protocol to assess the influence of typical components of MS, including a kMCT supplement, low-carbohydrate meal and meal timing, on blood ketones, glucose, insulin and free fatty acids (FFA). In one test, the effect of age was also investigated. Over the 8 h metabolic study day, two 10 g doses of the kMCT increased the plasma ketone response by 19% while reducing overall glycemia by 12% without altering insulin or FFA levels. Moreover, a single early meal (breakfast but no lunch) potentiated the ketogenic effect of MS over 8 h, compared to a single delayed meal (lunch but no breakfast). Age and the low carbohydrate meal did not affect the ketones response. We conclude that an 8-h test period can be used to assess metabolic changes during short-term MS. kMCT provide a robust short-term increase in ketones and might enhance the metabolic effectiveness of short-term or intermittent fasting as a component of MS.

## Introduction

Glucose is the brain's main energy substrate but during fasting or energy restriction, plasma ketones (acetoacetate [AcAc] and β-hydroxybutyrate [BHB]) increase and spare brain glucose utilization (1) in part due to their oxidative efficiency and competition with pyruvate to enter the citric acid cycle (2–4). Ketones uptake by the brain is directly proportional to their plasma concentrations (5) which increases as the liver produces ketones from free fatty acids (FFA) in response to low glucose and insulin induced during dietary energy or carbohydrate restriction (6). Mild to moderate nutritional ketonemia has long been known to be beneficial for the management of severe refractory epilepsy (7). Other neurotherapeutic uses of mild-moderate ketonemia are now being investigated, especially in conditions in which brain glucose utilization deteriorates, such as during aging (8–11). Eight carbon medium chain fatty acids cross the blood brain barrier (12) and have anapleurotic and signaling properties independent of ketonemia (13, 14).

Medium chain triglycerides of 8 and 10 carbons are ketogenic (kMCT) because of their rapid absorption and oxidation (15, 16). kMCT transiently raise plasma ketones to 0.5–1.0 mmol/L when taken as a 10–15 g dietary supplement at meals (10, 17, 18). Plasma ketones increase in a direct dose-response relationship to the oral dose of kMCT consumed (1). Emulsifying or consuming MCT without a meal increases their ketogenic effect (1, 18).

Metabolic switching (MS) involves planned recurrent dietary energy or carbohydrate restriction. Typically, the period of reduced energy/carbohydrate intake eliminates 1–2 meals/day or involves total fasting for one to several days at a time. An exercise regimen and exogenous ketogenic supplement may also be involved. During MS, glucose's contribution to the body's energy requirements declines and that of free fatty acid (FFA) and ketones increases (5). During periods of fasting and prolonged exercise, ketones are an important alternative fuel to glucose for the brain (19) and also peripherally (20). The mild to moderate ketonemia associated with MS may improve metabolic outcomes and perhaps brain health (21, 22).

Despite considerable research in animals, few experimental protocols for MS have been reported in humans. We have previously used an 8-h metabolic study protocol to compare the plasma ketone response after kMCT under different test conditions (18, 23, 24), so we used the same 8-h protocol to assess MS. In the present study, we hypothesized that three central aspects of MS, i.e., carbohydrate in the meal, kMCT, and meal timing, would augment the acute plasma ketone response during an 8-h test. Our objective was to use an 8-h study protocol to assess the ketogenic effect of a commercially available kMCT drink in relation to: (i) the dose of kMCT, (ii) carbohydrate content of the meal, (iii) meal timing (missing breakfast vs. missing lunch), and (iv) age. The kMCT dose was in the range we have used previously and permitted a doubling of the dose with minimal side-effects (10–20 g) in one of the experiments. The effect of meal timing was assessed by extending the overnight fast till mid-day and giving the first meal of the day as lunch. In addition to the plasma ketones, glucose, insulin and FFA were also measured.

## Methods

### Participants

Ethical approval for this study was obtained from the Research Ethics Committee of the Integrated University Health and Social Services of Eastern Townships—Sherbrooke University Hospital Center, which oversees all human research done at the Research Center on Aging (Sherbrooke, QC, Canada) and functions in accordance with the Declaration of Helsinki. All participants provided written informed consent prior to beginning the study and were recruited from June 2017 to May 2018. They underwent a screening visit, including the analysis of a blood sample collected after a 12 h overnight fast. Exclusion criteria included smoking, diabetes (fasting glucose >7.0 mmol/l and glycated hemoglobin >6.9%), strenuous aerobic exercise more than three times a week, untreated hypertension, dyslipidemia, and abnormal renal, liver, heart or thyroid function. Participants on medications known to affect triglyceride or carbohydrate metabolism (i.e., diuretics, beta-blockers, steroids, insulin sensitizers) were also excluded. This project was prospectively registered on ClinicalTrials.gov (NCT03830268).

### Metabolic Tests

The metabolic tests are summarized in Table 1. The kMCT drink was a 20% liquid emulsion (45 g MCT in 225 ml water plus an emulsifier and sweetener) containing 60% octanoic acid and 40% decanoic acid (BetaQuik® VitaFlo, Liverpool, UK). It has a caloric density of 1.7 kcal/mL.

**Table 1:** Summary of the metabolic interventions.

***N***	**Description**	**T0**		**T4**	
		**Meal**	**kMCT**	**Meal**	**kMCT**
6	Control baseline: no kMCT + meal at T0 (CTL)	X			
10	kMCT 10 g + meal at T0 (kMCT10)	X	X		X
10	kMCT 20 g + meal at T0 (kMCT20)	X	X		X
10	kMCT 10 g + LC meal at T0 (kMCT10-LC)	X	X		X
6	Metabolic switch, no kMCT (MS-CTL)			X	
8	Metabolic switch + kMCT 10 g (MS-kMCT10)		X	X	X
10	kMCT10 g in Older group + meal at T0 (kMCT Older)	X	X		X

*T0, time of first blood sample during metabolic study day; if a meal was taken at this time, it was immediately after the first blood sample. Blood samples were taken every 30 min after T0 for 8 h*.

*T4, time (h) after first blood sample: If a second meal was taken, it was taken immediately after the T4 blood sample*.

*kMCT, ketogenic medium chain triglyceride (10 g MCT in a 50 ml drink; see Methods)*.

### Experimental Design

The protocol involved six separate metabolic studies in young adults, and one comparing young and older adults aged 65 y old. On each metabolic study day, participants arrived at 7:30 a.m. after a 12 h overnight fast and a minimum of 24 h without alcohol intake. A forearm venous catheter was installed, and a baseline blood sample withdrawn. Participants then consumed the kMCT drink with or without a meal. The meal consisted of two pieces of toast with strawberry jam, a piece of cheese, and two scrambled eggs.

To simplify identifying the different tests and reporting the results, we will refer to MS as the tests with kMCT and the midday meal, as distinct from the tests with kMCT and the low carbohydrate (LC) or breakfast meal. To test if the post-prandial increase in blood glucose or insulin influenced changes in plasma ketones after kMCT, we provided an iso-caloric LC meal instead of the regular high carbohydrate meal (Table 2). The LC meal consisted of three pieces of cheese and three scrambled eggs providing 3 g instead of 49 g carbohydrate. A second dose of kMCT was given at noon, with or without a meal depending on the test.

**Table 2:** Meal and drink composition.

	**Composition**	**Calories**	**Fat (g)**	**Carbohydrate (g)**	**Protein (g)**	**Ratio**
Regular	2 eggs; whole wheat toast; strawberry jam; 1 slice of cheese	450	18.5	49	23.2	1:4
Low carbohydrate	3 eggs; 3 cheese slices	450	36	3	33	1:1
kMCT10	Medium-chain triglyceride; sucralose	88	10.5	0	0	1:0

*Macronutrient composition of the meal components: egg: 70 calories, 1 g carbohydrate, 6 g protein, 5 g fat; toast: 150 calories; 27 g carbohydrate; 6 g protein; 1.5 g fat; jam: 80 calories; 20 g carbohydrate; 0.2 protein; 0 g fat; cheese: 80 calories; 0 g carbohydrate; 5 g protein; 7 g fat*.

*Ratio = fat: carbohydrate + protein; kMCT10 = 10 g MCT in 50 ml of the kMCT drink*.

During the MS, participants had no meal or kMCT at T0 followed by the meal alone T4 (MS-CTL) or kMCT but no meal at T0 followed by the meal and kMCT at T4 (MS-kMCT). Water was available *ad libitum* throughout the test day. Blood samples were taken at baseline (immediately before the intervention) and every 30 min during 8 h with the first post-dose sample being taken 30 min after the kMCT drink was consumed.

### Plasma Metabolite Analyses

Blood samples were centrifuged at 2,846 g for 10 min at 4°C and plasma stored at −80°C until analyzed. Plasma BHB and AcAc were measured by an automated colorimetric assay. For AcAc, 25 μL of plasma was mixed with 330 μL of fresh reagent (Tris buffer, pH 7.0, 100 mmol/L, 20 mmol/L sodium oxamate; 0.15 mmol/L NADH and 1 U/mL β-hydroxybutyrate dehydrogenase [BHBDH]). For BHB, the reagent was Tris buffer (pH 9.0; 20 mM sodium oxamate, 1 mmol/L NAD, and 1 U/mL BHBDH). Tris, oxamic acid, DL-BHB sodium salt, Li-AcAc standard, and NAD were purchased from Sigma (St. Louis, MO, USA), NADH, from Roche (Mannheim, Germany), and BHBDH from Toyobo (Osaka, Japan). The change in absorbance at 340 nm between 15 and 120 s after the addition of the reagent was measured on an automated clinical chemistry analyzer (Dimension Xpand Plus; Siemens, Deerfield, IL, USA). The assay was calibrated with freshly diluted standards from frozen aliquots of a 10 mmol/L standard of Li-AcAc or DL-BHB sodium salt, which is stable at −20°C for 2 and 6 months, respectively. Calibrations and quality controls were performed for each assay to ensure the precision of the kits (coefficient of variation between tests 5 ± 1%). Where “ketones” are mentioned in the results, it refers to AcAc and BHB combined.

Plasma glucose and triglycerides (Siemens Medical Solutions USA, Inc., Deerfield, IL, USA) and FFA (Randox Laboratories Limited, West Virginia, USA) were analyzed using commercial kits. Plasma insulin was analyzed by enzyme-linked immunosorbent assay (Alpco Diagnostics Ltd., Salem, NH, USA) with a microplate reader (Victor multi-label plate reader 2030; Perkin Elmer, MA, USA). Glycated hemoglobin was measured by HPLC-723G7, a fully automated high-performance liquid chromatography instrument-reagent system (Tosoh Bioscience, King of Prussia, PA, USA). Plasma insulin was analyzed by ELISA (Alpco, Salem, NH, USA).

### Statistical Analysis

All statistical analyses were carried out using SPSS 23.0 software (SPSS Inc., Chicago, IL, USA). On metabolic test days, the test dose of kMCT was given at T0 or T4, so the half-day periods are reported as T0–4 and T4–8 to compare the different timing of the meal and kMCT drink. Areas-under-the-curve (AUC) from T0 to T4 and T4 to T8 were calculated according the trapezoid method (25). The Shapiro-Wilks test demonstrated that the plasma metabolite data were not normally distributed, so results were compared using the non-parametric Friedman's test, and the effect of the treatments was determined in each group using Wilcoxon's signed rank test. The Young and Older groups were treated as independent groups and compared using the Mann-Whitney test. Differences were considered statistically significant at *P* ≤ 0.05. Graphs were prepared using Prism version 6.0 (GraphPad Software Inc., San Diego, CA, USA). Preliminary analysis of the AcAc and BHB data did not show any major changes in this ratio across the treatments so these data are not reported. All results are given as the mean ± SD.

## Results

Six to ten young participants completed all seven metabolic tests (see Table 1 and figure legends). Baseline anthropometry and plasma metabolites corresponded to reference values from the Sherbrooke University Hospital Center (Sherbrooke, QC). No significant difference was found in anthropometric and plasma measures between the Young and Older groups, except for age, glucose and glycated hemoglobin (Table 3), and were within the normal range for age in both groups (26, 27). No adverse events related to the kMCT drink were reported.

**Table 3:** Participant characteristics.

	**Young**	**Older**	***P***
Gender (M:F)	4:6	4:6	
Age	28 ± 7	65 ± 6	<0.001
Body mass index (kg/m^2^)	24 ± 3	29 ± 5	>0.05
Ketones (μmol/L)	110 ± 71	87 ± 43	>0.05
Glucose (mmol/L)	4.8 ± 0.4	5.3 ± 0.6	0.009
Glycated hemoglobin (%)	5.1 ± 0.3	5.5 ± 0.2	0.008
Insulin (pmol/L)	46 ± 15	58 ± 18	>0.05
Triglycerides (mmol/L)	0.9 ± 0.5	1.2 ± 0.3	>0.05
Free fatty acids (mmol/L)	0.5 ± 0.2	0.5 ± 0.1	>0.05

*Values are mean ± SD; n = 10/group*.

*Ketones = acetoacetate + β-hydroxybutyrate combined*.

### Plasma Ketones

**Figure 1:**
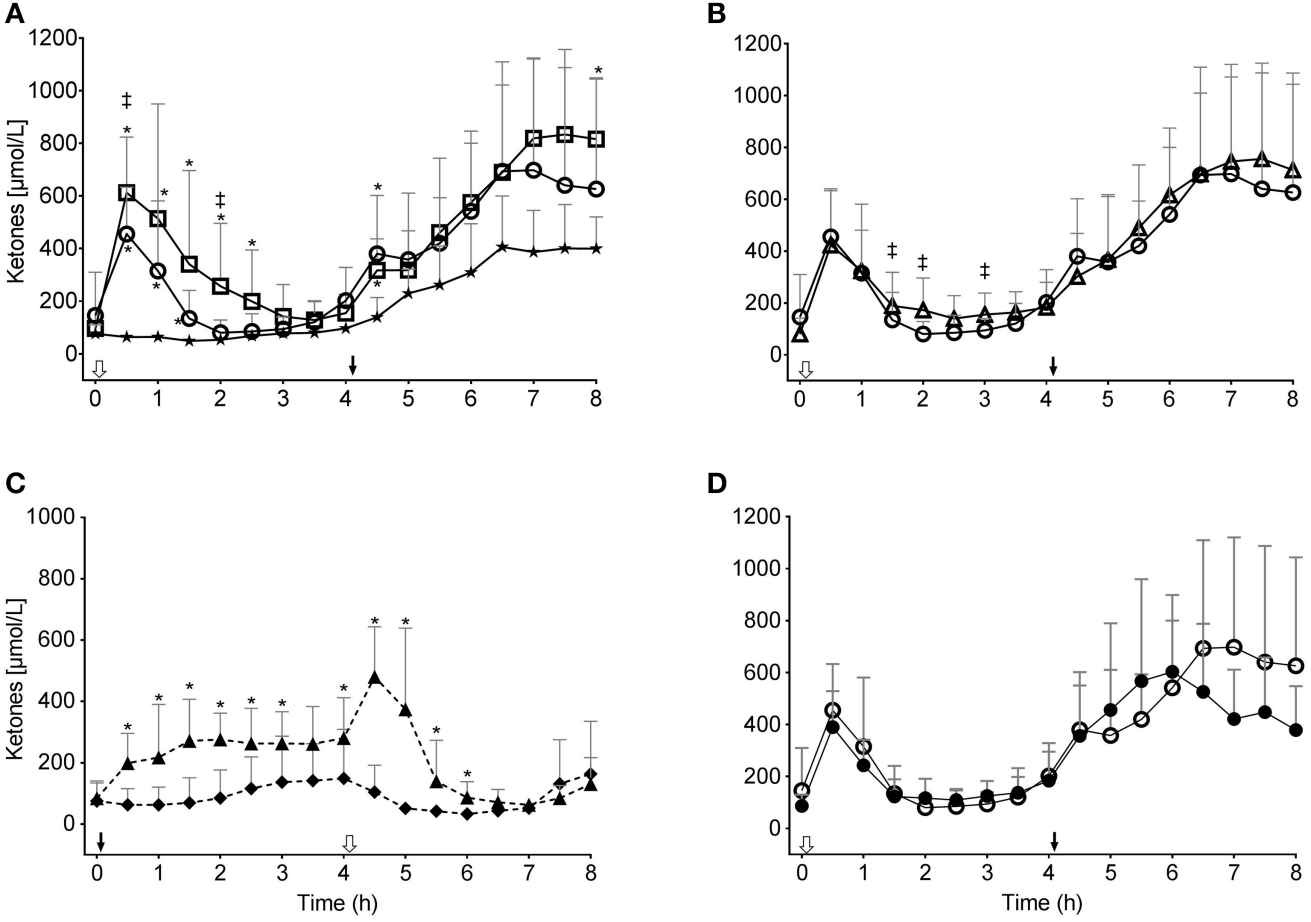
Plasma total ketone (β-hydroxybutyrate and acetoacetate combined) concentration during the tests: **(A)** without consuming kMCT (CTL, ★) or after 10 g (kMCT10, 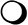) or 20 g (kMCT20, □); **(B)** after 10 g of kMCT with a carbohydrate-rich (kMCT10, 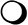) or low carbohydrate (kMCT10LC, Δ) meal; **(C)** after no meal or kMCT at T0 but a meal at T4 (MS-CTL; ⧫– –⧫) or no meal at T0 but a meal plus KMCT10 at T4 (MS-kMCT; ▴– –▴); **(D)** Older (•) vs. Young (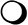) after KMCT10 at T0. The black arrow indicates when the meal plus kMCT were consumed and the white arrow indicates when kMCT alone was consumed without the accompanying meal. Values are presented as mean ± SD (*n* = 6–10). ^*^Difference with CTL; ^‡^Difference between the different test conditions (*P* < 0.05).

**Figure 2:**
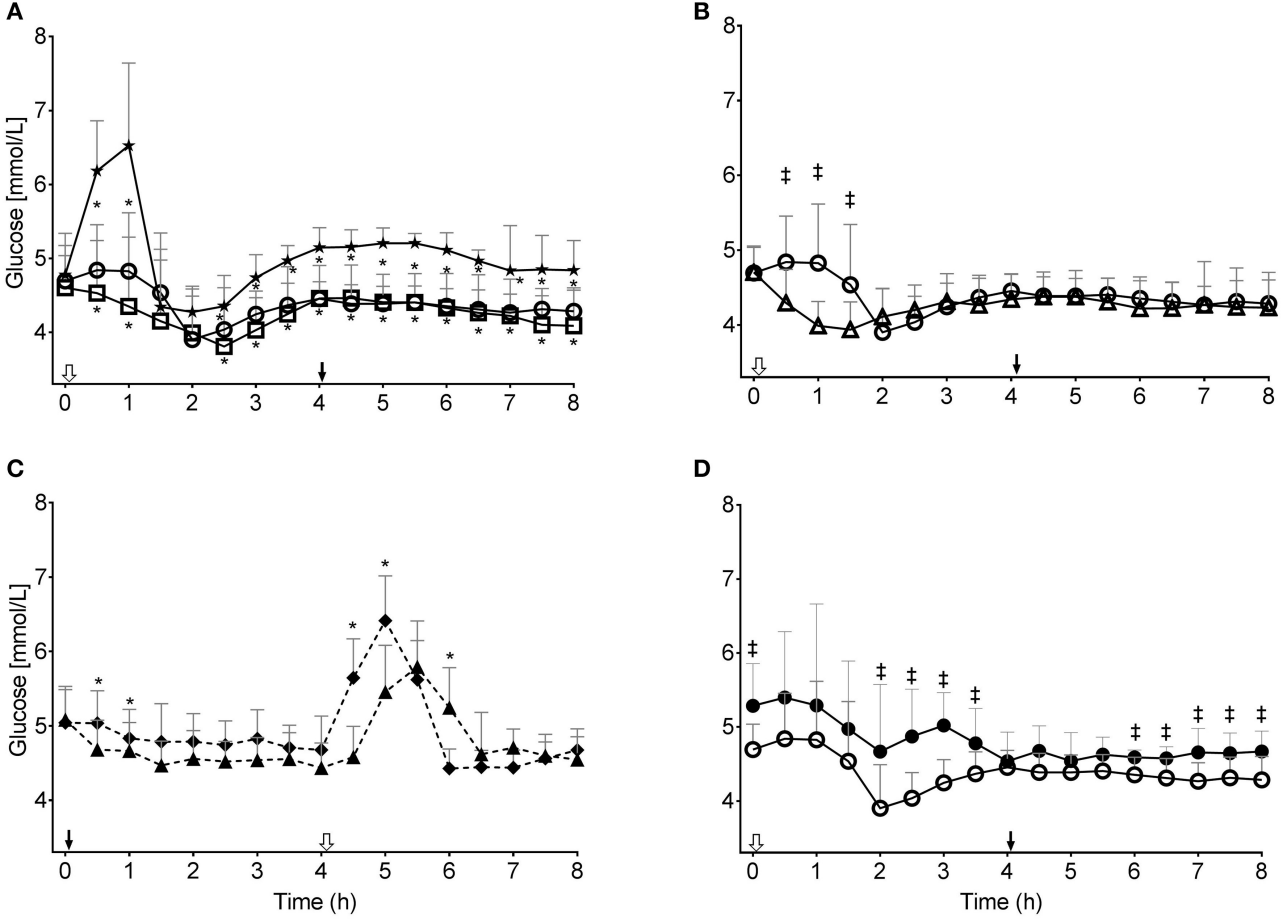
Plasma glucose concentration during the tests: **(A)** without consuming kMCT (CTL, ★) or after 10 g of kMCT (kMCT10, 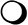) or 20 g of kMCT (kMCT20, □); **(B)** after 10 g of kMCT with a carbohydrate-rich (kMCT10, 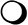) or low carbohydrate (kMCT10LC, Δ) meal; **(C)** after no meal or kMCT at T0 but a meal at T4 (MS-CTL; ⧫– –⧫) or no meal at T0 but a meal plus kMCT10 at T4 (MS-kMCT; ▴– –▴); **(D)** Older (•) vs. Young (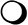) after kMCT10 at T0. The black arrow indicates when the meal plus kMCT were consumed and the white arrow indicates when kMCT alone was consumed without the accompanying meal. Values are presented as mean ± SD (*n* = 6–10). *Difference with CTL; ^‡^Difference between the different test conditions (*P* < 0.05).

Oral intake of a 10 or 20 g dose of kMCT (kMCT10 and kMCT20, respectively) at T0 significantly elevated plasma ketones over about 3 h in a dose-dependent fashion compared to the CTL (Figure 1A). The ketone C_max_ was 455 ± 125 and 612 ± 160 μmol/L, for kMCT10 and kMCT20, respectively (*P* = 0.028). During the CTL test, plasma ketone levels remained between 49 and 97 μmol/L between T0 and T4 but increased to about 400 μmol/L from T6.5 to T8 (Figure 1A). The T0–4 AUCs for kMCT10 and kMCT20 were both significantly higher than the CTL test (*P* = 0.028; Figure 2). During the MS test, the second dose of kMCT raised plasma ketones over baseline between T4 and T8. The increase in plasma ketones after kMCT10 was essentially independent of the carbohydrate content from the meal between T0 and T4 (Figure 1B, Table 4).

**Table 4:** Areas-under-the-curve for total ketones (μmol h/L; β-hydroxybutyrate + acetoacetate) during the first 4 h (T0–T4) or the second 4 h (T4–T8) of the metabolic study days (see Table 1 for legend).

	**T0–T4**		***P*-value**
CTL vs. kMCT10	271 ± 45	730 ± 260	0.028
CTL vs. kMCT20	271 ± 45	*1, 159*±736	0.028
kMCT10 vs. kMCT20	730 ± 260	*1, 159*±736	0.059
kMCT10 vs. kMCT10LC	730 ± 260	780 ± 436	0.721
MS-CTL vs. MS-kMCT10	394 ± 379	967 ± 327	0.046
kMCT10 Vs. Older	730 ± 260	695 ± 130	0.853
	**T4–T8**		***P*****-value**
CTL vs. kMCT10	*1, 191*±502	*2, 023*±*1, 131*	0.116
CTL vs. kMCT20	*1, 191*±502	*2, 263*±795	0.028
kMCT10 vs. kMCT20	*2, 023*±1, 131	*2, 263*±795	0.508
kMCT10 vs. kMCT10LC	*2, 023*±*1, 131*	*1, 975*±*1, 027*	0.959
MS-CTL vs. MS-kMCT10	307 ± 194	753 ± 326	0.046
kMCT10 Vs. Older	*2, 023*±*1, 131*	*1, 852*±635	0.796

*Values are presented as mean ± SD*.

The MS-CTL test showed constantly low blood ketones between 33 and 150 μmol/L over 8 h (Figure 1C) and did not display the same ketone increase between T4-T8 as seen with the CTL baseline (Figure 1A). Without the meal at T0, kMCT10 produced a more prolonged increase in ketones up to the meal taken at T4 (MS-kMCT). The second 10 g dose of kMCT in the MS-kMCT10 test transiently raised ketones followed by a rapid decrease to baseline like the change in ketones after the first dose of kMCT. There was no significant difference in plasma ketones in the Young compared to Older group after kMCT10 (Figure 1D, Table 4).

### Plasma Glucose

**Figure 3:**
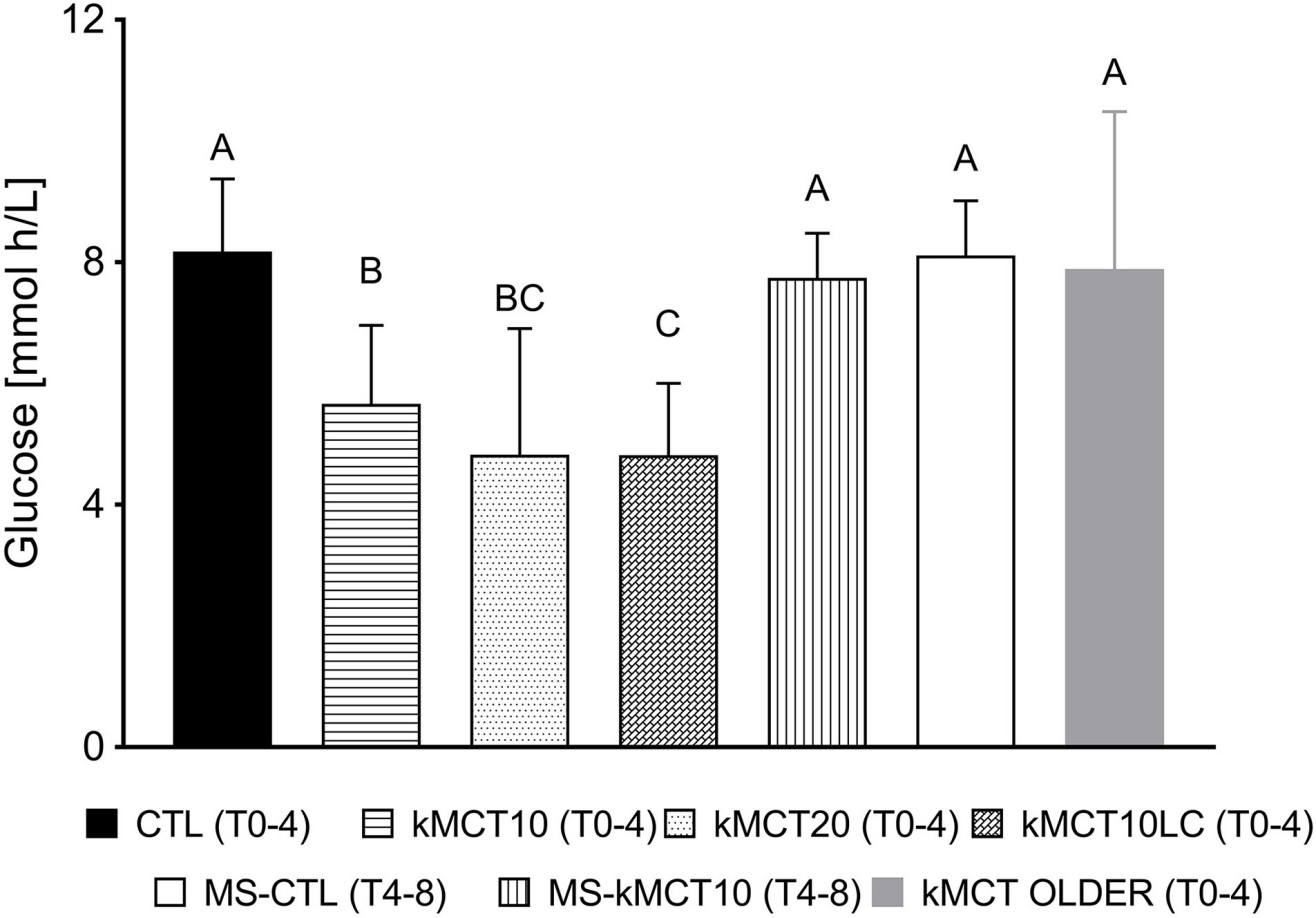
Area-under-the-curve (mmoLh/L) for the post-prandial plasma glucose response at T0-T4 without consuming kMCT (CTL), after 10 g of kMCT (kMCT10), with no meal at T0 but a meal at T4 without kMCT (MS-CTL) and no meal at T0 but a meal at T4 with 10 g of kMCT (MS-kMCT10). The time period of the area-under-the-curve for each bar is shown in brackets. Values are presented as mean ± SD (*n* = 6–10). Means without a common letter differ significantly (A < B); *P* < 0.05.

The post-prandial plasma glucose response to a standard meal showed that kMCT10 and kMCT20 reduced glucose values at most time points compared to CTL without a difference between the plasma glucose response to kMCT10 vs. kMCT20 (*P* < 0.05), and also unaffected by the second dose of kMCT taken at T4 (Figure 2A). The AUC for plasma glucose between T0 and T4 was reduced by 30–42% by concomitant consumption of kMCT10 (Figure 3) or kMCT20. During the MS-kMCT10 test, post-prandial glucose between T4 and T8 was significantly lower than MS-CTL at T4.5 and T5 (*P* < 0.017), and the T_max_ was 30 min later (T 5.5 vs. T5; Figure 2C). The post-prandial glucose response was lower over the first 4 h when the kMCT10 was taken with a LC meal (Figures 2B, 3). The post-prandial glucose response to the standard meal at T0 was significantly higher in the Older vs. the Young group at T0 (fasting), from T2 to T3.5 and from T6 to T8 (Figure 2D; *P* < 0.05), with a correspondingly higher T0 to T4 AUC in the Older group as well (Figure 3).

### Plasma Insulin

**Figure 4:**
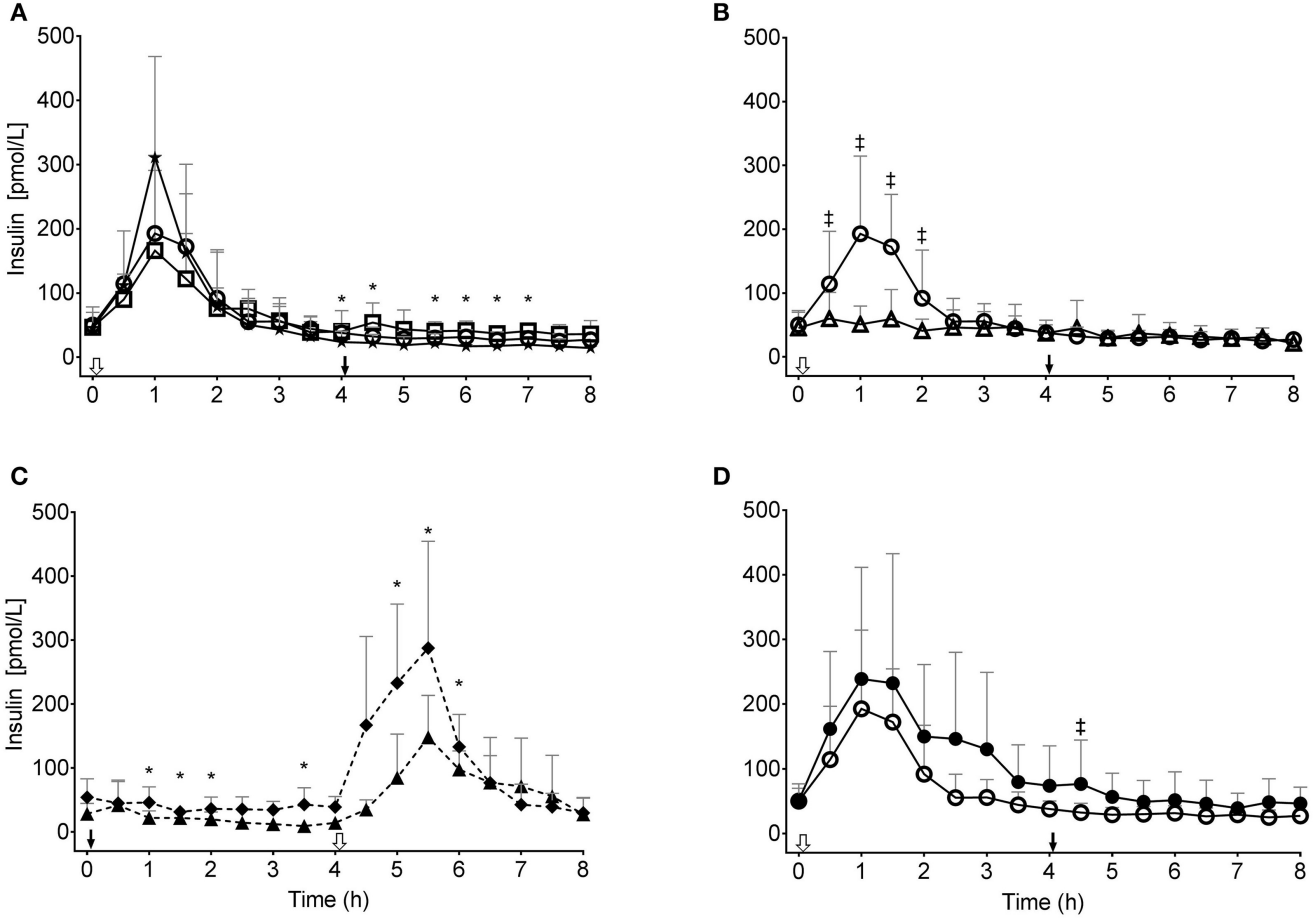
Plasma insulin concentration during the tests: **(A)** without consuming kMCT (CTL, ★) or after 10 g or 20 g of kMCT (kMCT10, 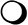; kMCT20, □); **(B)** after 10 g of kMCT with a carbohydrate-rich (kMCT10, 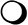) or low carbohydrate (kMCT10LC, Δ) meal; **(C)** after no meal or kMCT at T0 but a meal at T4 (MS-CTL;⧫– –⧫), or no meal at T0 but a meal plus kMCT10 at T4 (MS-kMCT; ▴– –▴; **(D)** Older (•) vs. Young (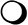) after kMCT10 at T0. The black arrow indicates when the meal plus kMCT were consumed and the white arrow indicates when kMCT alone was consumed without the accompanying meal. Values are presented as mean ± SD (*n* = 6–10). *Difference with CTL; ^‡^Difference between the different test conditions (*P* < 0.05).

During the CTL, kMCT10 and KkMCT20 tests, plasma insulin peaked at 311 ± 134, 193 ± 93, and 166 ± 98 pmol/L (all at T1), respectively, with no significant difference across the three tests (Figure 4A). The insulin response to kMCT20 was significantly higher at most T4–8 time points compared to CTL (*P* < 0.046). During kMCT10LC, plasma insulin was significantly lower than either CTL or kMCT10 at several time points (*P* < 0.038; Figure 4B). During the two MS tests, plasma insulin peaked at 287 ± 80 (T5.5) and at 148 ± 52 pmol/L (T5.5; *P* < 0.046; Figure 4C). On the kMCT10 test, plasma insulin only differed between the Young and Older groups at T4.5 (*P* < 0.05; Figure 4D). Insulin AUC did not differ between tests (data not shown).

### Plasma Free Fatty Acids

**Figure 5:**
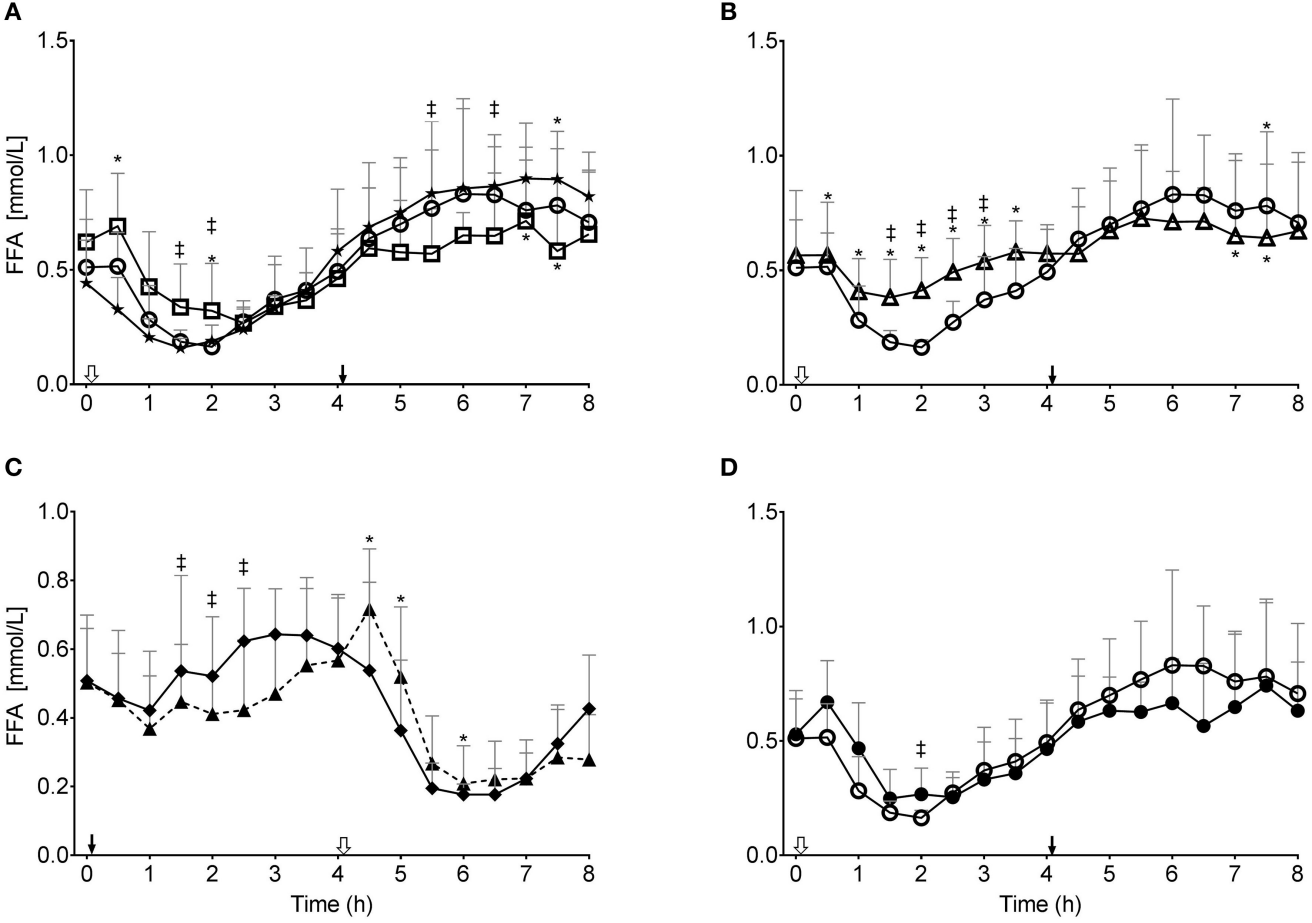
Plasma free fatty acid (FFA) concentration during the tests: **(A)** without consuming kMCT (CTL, ★) or after 10 g or 20 g of kMCT (kMCT10, 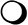; kMCT20, □); **(B)** after 10 g of kMCT with a carbohydrate-rich (kMCT0, 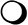) or low carbohydrate (kMCT10LC, Δ) meal; **(C)** after no meal or kMCT at T0 but a meal at T4 (MS-CTL; ⧫– –⧫) or no meal at T0 but a meal plus kMCT10 at T4 (MS-kMCT; ▴– –▴); **(D)** Older (•) vs. Young (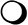) after kMCT10 at T0. The black arrow indicates when the meal plus kMCT were consumed and the white arrow indicates when kMCT alone was consumed without the accompanying meal. Values are presented as mean ± SD (*n* = 6–10). *Difference with CTL; ^‡^Difference between the different test conditions (*P* < 0.05).

The plasma FFA response differed very little from CTL after either dose of kMCT (Figure 5A), with the MS (Figure 5C) or in the Older group (Figure 5D). There was a significantly lower trough and peak plasma FFA after the LC meal (Figure 5B).

### Metabolic Changes Over 8 h

**Figure 6:**
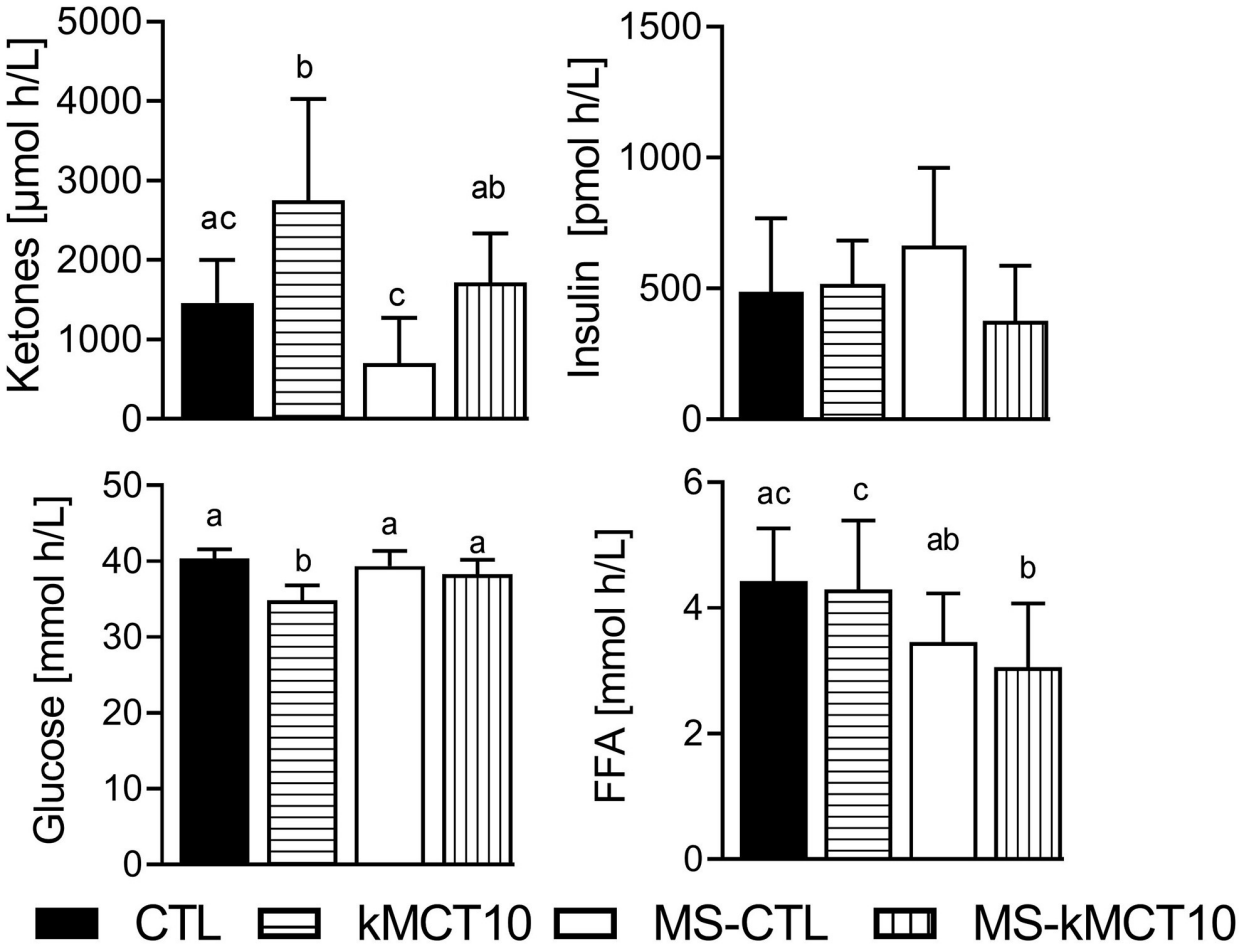
Area-under-the-curve for the plasma ketone, glucose, insulin and free fatty acid (FFA) response over the full 8 h of the metabolic study day without consuming kMCT (CTL), after 10 g of kMCT (kMCT10), with no meal at T0 but a meal at T4 without kMCT (MS-CTL) or with no meal at T0 but a meal at T4 with 10 g of kMCT (MS-kMCT10). Values are presented as mean ± SD (*n* = 6–10). Means without a common letter differ significantly (A < B); *P* < 0.05.

To evaluate the overall impact of the main metabolic tests (kMCT, MS) on plasma ketone, glucose, insulin and FFA, AUCs were compared for the full T0-T8 period (Figure 6). kMCT10 induced a significantly lower glycemic response, no change in ketones or insulin response, and higher FFA response when compared with MS kMCT10.

## Discussion

The main observation was that over the 8 h test period, two 10 g doses of kMCT increased the plasma ketone response by 88% while reducing glycemic response by 12%, without altering insulin or FFA levels. After an overnight fast, an early meal (breakfast) was more effective at potentiating the ketogenic effect of MS over 8 h, compared to a delayed meal (lunch) without breakfast. When compared to our standard high carbohydrate meal, a LC meal lowered plasma glucose and insulin but had similar plasma ketone response from kMCT.

Like other kMCT preparations reported previously (18, 28), the kMCT drink used here rapidly increased plasma ketones with an equivalent ketogenic effect observed whether it was taken with a meal in the morning or at midday (Figure 1C). The ketonemia induced by kMCT lasted 4–5 h so projected over a typical intake of three meals/day, would result in mild ketosis for at least 12–15 h/d. The ketogenic response to kMCT20 was higher than for kMCT10 in the 30 min after consuming the drink (Figure 1A), but the 20 g dose did not actually double the ketogenic response. These results confirm that although the plasma ketone response increases with the dose of MCT taken orally over a large range (10–70 g) (5), the response is not necessarily linear over a small range (10 to 20 g) and may well-depend on how it is provided with the volume of the drink playing a role (29). The timing of macronutrient absorption varies with energy density, volume and digestibility of meal and differs significantly between liquid and solid foods (30, 31).

Few experimental protocols for MS have been reported in humans. Our MS protocol described here with a first dose of kMCT in the morning without breakfast and a second dose of kMCT at noon with a meal increased the plasma ketone response by 145%. Plasma glucose changes were inversely related to those of ketones suggesting that MS conditions were being met albeit over only 8 h making this protocol relatively simple to test in other study conditions.

During healthy aging, fasting plasma glucose tends to be higher and brain glucose uptake declines but brain ketone uptake does not change (32, 33). After consuming a ketogenic drink containing kMCT, older people in good health have previously been shown to have a similar plasma ketone response and oxidize carbon-13-labeled BHB at the same rate as middle-aged or young adults (34). Here we confirm that the plasma ketone response induced with kMCT while consuming a high-carbohydrate meal was unaffected by aging and this despite the usual trend toward higher plasma glucose and mild insulin resistance in older people (Figures 1–3) (33, 35). A daily 30 g dose of MCT taken for 6 months was not associated with more side-effects than a high oleic acid placebo and did not change compliance or cardiovascular risk markers in an older population (10), suggesting good long-term acceptability, safety and validity of the 10 and 20 g doses of kMCT tested here.

This study had some limitations. First, the metabolic response to a single dose of kMCT may not be extrapolatable to longer term consumption of MCT. However, one report showed that consumption of a ketogenic MCT drink for 2–3 months did not significantly change the plasma ketone response compared to before starting the MCT (11). Second, we did not have a CTL metabolic test for the Older group, but the plasma ketone response of older people is very similar to that in young people (34). Third, although adequately powered for a significant effect of the dose of MCT on plasma ketones in young adults, the number of participants was still small making the study vulnerable to type II error.

We conclude that an 8-h MS protocol involving two doses of kMCT, the first taken with breakfast and the second without lunch, increases the plasma ketone response independent of carbohydrate in the meal or age, and without affecting the insulin or FFA level. Thus, a kMCT drink can provide a robust short-term increase in ketones under various feeding conditions and might enhance the metabolic effectiveness of short-term MS or intermittent fasting.

## Data Availability Statement

The datasets generated for this study are available on request to the corresponding author.

## Ethics Statement

The studies involving human participants were reviewed and approved by Research Ethics Committee of the Integrated University Health and Social Services of Eastern Townships—Sherbrooke University Hospital Center. The patients/participants provided their written informed consent to participate in this study.

## Author Contributions

SC, BC, C-AC, and MF designed the study and CV, VS-P, C-AC, and MF conducted it. CV, VS-P, and SC analyzed the data. All authors contributed to the final article.

### Conflict of Interest

SC acted as an external consultant to Nestlé, Bulletproof and Accera and received research funding and/or research materials from Abitec and Nestlé. SC is founder and CEO of Senotec Ltd. The remaining authors declare that the research was conducted in the absence of any commercial or financial relationships that could be construed as a potential conflict of interest.
